# High diversity of arthropod colour vision: from genes to ecology

**DOI:** 10.1098/rstb.2021.0273

**Published:** 2022-10-24

**Authors:** Ayse Yilmaz, Natalie Hempel de Ibarra, Almut Kelber

**Affiliations:** ^1^ Department of Biology - Functional Zoology, Lund University, Lund 22362, Sweden; ^2^ School of Psychology, University of Exeter, Exeter EX4 4QG, UK

**Keywords:** colour vision, visual pigments, photoreceptors, sensory biology, visual ecology, colour ecology

## Abstract

Colour vision allows animals to use the information contained in the spectrum of light to control important behavioural decisions such as selection of habitats, food or mates. Among arthropods, the largest animal phylum, we find completely colour-blind species as well as species with up to 40 different opsin genes or more than 10 spectral types of photoreceptors, we find a large diversity of optical methods shaping spectral sensitivity, we find eyes with different colour vision systems looking into the dorsal and ventral hemisphere, and species in which males and females see the world in different colours. The behavioural use of colour vision shows an equally astonishing diversity. Only the neural mechanisms underlying this sensory ability seems surprisingly conserved—not only within the phylum, but even between arthropods and the other well-studied phylum, chordates. The papers in this special issue allow a glimpse into the colourful world of arthropod colour vision, and besides giving an overview this introduction highlights how much more research is needed to fill in the many missing pieces of this large puzzle.

This article is part of the theme issue ‘Understanding colour vision: molecular, physiological, neuronal and behavioural studies in arthropods’.

## 1. Introduction

Arthropods are the largest phylum in the animal kingdom, with respect to the number of species (an estimated 7 million species of terrestrial arthropods, [1]) and, despite recent declines, also the number of individual animals roaming our planet. Arthropods inhabit any habitat on the Earth, from the deep sea to mountain tops, from the arctic regions to the tropics, in air and water. Their sophisticated senses help them to find habitats, food, mates and nests, and here arthropods set another record: their colour vision shows by far the highest diversity among all animal phyla. Among arthropods we find species which are completely colour-blind, but also the animals with the most complex colour vision systems. This high diversity extends to all functional levels from the molecular opsin-based visual pigments, the physical and anatomical adaptations determining spectral sensitivity of photoreceptor cells and eyes to colour-guided behaviours and ecology of colour vision.

Colour vision allows animals to extract information from the spectral composition of light reaching the eyes, by comparing the number of photons in different spectral ranges absorbed by photoreceptors differing in spectral sensitivity and subsequent neural processing. The dimensionality of colour vision is defined by the number of receptor types that provide input into colour processing. As in vertebrates, di-, tri- and tetrachromatic colour vision (based on two, three and four receptor types, respectively) has been described in arthropods. Spectral information—seen as colour—is found everywhere. The sky changes colour over the day [2] and differs in colour between its solar and antisolar half (e.g. [3]). Water bodies change colour with depth and scattering particles (e.g. [4]), the predominantly green colour of vegetation stems largely from the absorbance spectrum of chlorophyll and secondary metabolites, while bird feathers, the cuticle of arthropods, wing scales of insects and integuments of other animals can produce colour by combining structural mechanisms and pigments (e.g. [5–7]). Finally, colourful flowers and other plant structures stand out from the green foliage (e.g. [8–10]).

As early as in the seventh century, Sprengel [11] had noticed that the flower colours were aimed at attracting pollinating insects rather than pleasing the human eye. The first clear evidence for colour vision in an arthropod—the water-flea *Daphnia*—was presented by Lubbock [12], but this sensory ability was still discussed fiercely in the 1910s (e.g. [13]). More than 100 years later, we have gained a solid body of knowledge on the topic. Among arthropods, insects remain the best-studied clade (e.g. [14,15]), but spectral sensitivity, colour vision and related questions have also been investigated in chelicerata (e.g. [16–18]) and crustaceans (e.g. [19,20]). This special issue aims to present the current state of knowledge on arthropod colour vision by combining reviews, new research articles and a methods paper reporting a novel tool for generating colour stimuli. Given the large volume of recent research, it can only give a glimpse into the vast diversity of arthropod colour vision. The contributions highlight important advances made in recent years and point towards areas in which knowledge is still sparse, ranging from topics such as opsin gene diversity and opsin gene evolution, receptor sensitivity and physiology, neuronal processing to behavioural and ecological studies including the use of colour vision in navigation and communication.

## 2. Diversity of arthropods opsins

Living organisms have evolved different types of photosensitive pigments, including, for instance, phytochromes and cryptochromes. However, animal vision is almost exclusively (but see [21]) based on photopigments in which an opsin—a light-sensitive G-protein coupled receptor—binds a vitamin A-derived chromophore. Of the large family of opsin proteins, arthropods mainly express the visual Gq coupled rhabdomeric or r-opsins in the rhabdomeric photoreceptors of their eyes [22], while vertebrates express ciliary or c-opsins in their rods and cones. Whereas the common ancestor of Tardigarda is reported to only have had one visual r-opsin [23], and a similar situation is found in the Onychophora [24], the ancestral arthropod supposedly possessed five visual r-opsins, two sensitive to light of long wavelengths (green-sensitive; LWS1 and LWS2), two sensitive to light of medium wavelengths (blue-sensitive; MWS1 and MWS2) and one to light of short-wavelengths (ultraviolet-sensitive; SWS or sometimes named UVS) [25]. In addition to the visual r-opsins, arthropods have other opsins in their genomes, including pteropsin expressed in the brain [26] and some, specifically among chelicerates and possibly dragonflies, also express peropsins/retinal G-protein coupled receptors in eyes [25,27].

Arthropod opsin evolution has been very dynamic [25], with repeated losses and gene duplications leading to the complex pattern we see today. Groups such as deep-sea crustaceans are using a single opsin while most clades have some type of colour vision, with extremes of up to 40 r-opsin genes in the genome, as in dragonflies [15,28], or up to 15 opsins expressed in the eyes, as in some stomatopod crustaceans [20].

Among Chelicerata, opsins diversity of horseshoe crabs [27] and some spiders [29] have been studied. Pancrustaceans have been covered well by Henze & Oakley [25], but Palecanda *et al.* [19] present the most comprehensive study on opsins in crustaceans to date and report some clades to have only one or two visual opsins (r-opsins) while others have multiple gene duplications specifically of LWS and MWS opsins. A detailed report on the best studied and likely most diverse clade among crustaceans, the stomatopods, is presented by Cronin *et al.* [20]. Among insects, some orders have been heavily studied, for instance Hymenoptera (for ants, see [30]) and Lepidoptera [31], while for others, including the large orders of Diptera (flies) and Coleoptera (beetles), knowledge is still scarce [14]. McCulloch *et al*. [15] give an overview of the present state of knowledge on insect opsins.

Owing to the advancement of sequencing methods, the number of known visual opsins has increased dramatically for arthropods, but characterization of their spectral sensitivities has lagged behind. It has also become evident that opsins belonging to the same gene family can code for pigments with quite different sensitivities (e.g. [31]). The LWS opsins have been the most interesting case recently; while ancestral genes code for green-sensitive pigments, with peak absorbance somewhere between 500 and 540 nm, tuning mechanisms allowing for long-wavelength shifts and thus leading to red-sensitive opsins have been described in stomatopods [32] and various butterflies (e.g. [33,34]). Lienard *et al*. [31] discuss methods suitable to gain even further insights into spectral tuning and the evolution of opsin pigments in arthropods.

## 3. Diversity of opsin expression patterns

The expression patterns of opsin genes in eyes and photoreceptors add another dimension to the diversity. Different opsin genes can be expressed at different times during development (e.g. in odonates: [28]; in tardigrades, [23]), in different eye types (e.g. insect ocelli and compound eyes; see [25]) and even different regions of compound eyes (e.g. odonates: [28]). Colour vision is only possible if opsins are expressed simultaneously, in the same eyes (unless signals from different eyes influence behavioural choices), and in different photoreceptors, which, however, share the same visual field.

Among arthropods, both simple and compound eyes are found, and many clades have multiple eyes, such as ocelli and compound eyes in insects [25,35] or the four eye pairs of spiders [29]. Species with multiple eye types often express different opsins in different eyes, which make their use for colour vision highly unlikely. Similarly, two or several opsins expressed in the same photoreceptors, do only contribute to colour vision if this receptor's signal is compared to that of another type. Horseshoe crabs, for instance, express different subsets of their 18 opsins (including peropsin) in their lateral compound eyes and the two median eyes. Most photoreceptors co-express multiple LWS (and/or MWS) opsins, and each eye type, as well as light-sensitive segmental ganglia also contain UVS opsin [27], and thus, fulfil the basic precondition for colour vision. In spiders, the expression of LWS and UVS opsins can differ between eye types; among the species investigated by Morehouse *et al.* [29] the majority express two LWS and one (or two) UVS, often in different retinal layers, but they also found spiders in which no UVS opsin was apparent, and which thus may not have the potential for colour vision.

In many crustaceans, larval eyes express only one opsin, but in some, UV and LWS opsins are found, allowing for colour vision [36]. Many insect clades also have multiple opsin types expressed in larval eyes [37–39], and some also express two opsins (LWS and UVS) in the ocelli, allowing for spectral comparisons [40]. While the same UVS opsin is expressed in ocelli and compound eyes, the LWS expressed opsins can differ between the eye types [25].

In insects, colour vision is mainly based on the compound eyes, and the number of expressed opsins differs dramatically between species. Many species express three types of opsins in the compound eyes (one LWS, one MWS and one UVS, for a review, see [14]) allowing for trichromatic colour vision, but in some clades the numbers can be much higher. McCulloch *et al.* [15] summarize what is presently known on expression patterns and their development in the best-studied model *Drosophila* and other insects. Since the homology of fly and butterfly expression patterns has been recognized [41,42], it has become apparent that findings on *Drosophila* can often be generalized to other insects. Many insect compound eyes have complex opsin expression patterns, with two (flies) or three (Hymenoptera, Lepidoptera, Hemiptera) or even more (some Lepidoptera, see [43,44]) types of ommatidia arranged in a random mosaic, each expressing a different subset of opsins in their seven to nine photoreceptors [42,45]. While the control of mosaics with two or three ommatidial types by stochastic expression of a transcription factor (*spineless*) is now understood [41,42,46], additional mechanisms still await detection [43].

On top of this, the compound eyes of many insects and crustaceans show some form of regionalization of opsin gene expression. Some stomatopod crustaceans, for instance, express two opsins in the ommatidia of the large ventral and dorsal hemispheres of their eyes, but a large number in photoreceptors of the midband ommatidial rows, and moreover, different subsets of opsins in each of the six midband rows (comprehensively reviewed by Cronin *et al*. [20]). Dragonflies express different subsets of opsins not only between the larval and adult eyes, but also between the dorsal and ventral halves of their eyes [28]. Many butterflies, and also mosquitos [47] and crickets ([24], and reviewed by McCulloch *et al*. [15]) have regionalized opsin expression in the eyes. Moreover, butterflies of many species express different opsins between males and females [44,48], leading to different and complex colour vision systems in both sexes.

Co-expression of two visual r-opsins in the same photoreceptor has first been reported in crustaceans [49], and since, been found quite often, for instance in some butterfly eyes (e.g. [50]). It can influence and broaden the spectral sensitivity of the receptor, but does not otherwise contribute to colour vision. By contrast, co-expression is one reason why the dimensionality of colour vision is often lower than the number of opsins in a genome may suggest.

Besides the co-expression of visual r-opsins and probably small amounts of peropsin in some chelicerate eyes [27,29], Koyanagi *et al.* [51] report on the co-expression of opn3, an opsin belonging to the c-opsins, with op9, a blue-sensitive visual r-opsin, in one type of photoreceptor in the eyes of mosquitos and *Drosophila*. The effect of this co-expression of two opsins with different phototransduction cascades for vision, and specifically, colour vision, is yet unknown.

## 4. Diversity of optical mechanisms tuning the spectral sensitivity of photoreceptors

In addition to opsin expression, a number of optical mechanisms that can strongly modify both the spectral sensitivity of photoreceptors in an eye and the dimensionality of colour vision have been described in arthropods (see [14,30,52]).

Opsin-based pigments have broad spectral sensitivities, which are overlapping to a large extent, specifically in the UV range, where all pigments, independent of the main (alpha) peak sensitivity, have a beta peak (see the template functions by Stavenga *et al*. [53] and Govardovskii *et al*. [54]). The most basic mechanisms of influencing spectral sensitivity is the spatial organization of retinal photoreceptors. Long rhabdoms of photoreceptors have broader sensitivity than the pigment, owing to self-screening, at least in low light intensities. By contrast, if the rhabdoms of photoreceptors in a single ommatidium are fused as, for instance, in bees, they act as lateral filters for each other, thereby narrowing spectral sensitivity ([55]; for illustrations of both mechanisms, see [56]). Many arthropod eyes—both simple eyes and compound eyes—have tiered retinae, in which the rhabdoms are stacked in two or more layers. In these retinae, the more distal rhabdoms filter the light reaching rhabdoms in proximal and basal layers, again narrowing sensitivity. As SWS-sensitive receptors commonly contribute their rhabdoms to the most distal layer, this structure often removes the UV-sensitivity of the opsin-based MWS and LWS pigments expressed in the more proximal retinal layers. Layered retinae have been described in many crustaceans [20] and diverse insects, including, among others, odonates (e.g. [57]), flies [15] and butterflies [43,44,48] and the principal eyes of jumping spiders [18,58].

Additional filter mechanisms have been found on all levels of the optical systems of arthropod eyes [14]. Absorbing and fluorescing pigments have been found in the cornea (e.g. in flies: [59]; reviewed by Stavenga [60]) and crystalline cone (e.g. stomatopods: [61]; reviewed by [20]), where some of them influence spectral sensitivity. By contrast, López Reyes *et al.* [62] report that the function of fluorescing filters observed in thysanopteran insects (thrips) still awaits understanding. Filter pigments can also be placed more proximally in the light path, affecting only part of the photoreceptors. In a salticid spider, which expresses two visual pigments (UVS and LWS) in the retina, a red photostable pigment is placed in the light path to a subset of the photoreceptors with the LWS pigment, shifting its peak sensitivity from green to red, and thus creating a trichromatic visual system based on only two opsin-based pigments [18]. In stomatopods, additional filter pigments between rhabdom tiers act as long-pass filters for the proximal tiers, narrowing and shifting the peak sensitivity of photoreceptors to longer wavelengths. Maybe most surprisingly, these pigments change flexibly and over only a few days depending on the experienced light environment [20].

In insects such as many butterflies, filter pigments are placed in close vicinity to the rhabdoms which are narrow enough to act as light guides, thus filtering the light and again, changing the spectral properties of underlying receptors (e.g. [50,63], reviewed by Arikawa [48]). Pirih *et al.* [43] and Ilić *et al.* [44] give additional examples for this mechanism. Finally, in flies, sensitizing pigments in the receptors broaden the spectral sensitivity to shorter wavelengths (e.g. [64]).

## 5. Neural coding of colour is conserved

The diversity of visual pigments, photoreceptor types and ommatidial structures that underlies colour vision systems in arthropods is now well understood, but it is also evident that not all opsin genes and photoreceptors contribute to colour vision. Until recently, very little was known on the neural circuitry that analyses the signals from different receptor types in the small arthropod brains [42] and extracts the spectral information relevant to guide behavioural reactions. Colour processing requires inhibitory interactions between neurons with different spectral tuning (e.g. [65]), and it is now understood that colour coding involves multiple stages [66] including inhibitory interactions already at the photoreceptor stage.

In photoreceptors of butterflies spectrally opponent signals had been recorded already by Matić *et al.* [67]. Yet only recent work in flies and butterflies (e.g. [43,68,69]) has clearly proved that direct mutual inhibition between different spectral types of photoreceptors crucially contributes to insect colour coding. Flies have two spectral types of ommatidia (named P and Y) in which photoreceptors (named R7 and R8) express different opsins [70]. Two types of inhibition are critically involved in colour processing. In addition to direct inhibition between the receptors R7 and R8 in the same ommatidium, an interneuron mediates inhibition between these receptors in one ommatidial type and the equivalent receptors in adjacent ommatidia of the other type [68,71,72]. In butterflies with three ommatidial types [48]), ultraviolet-sensitive (SWS) receptors and blue-sensitive (MWS) receptors within the same ommatidium receive inhibitory input from green-sensitive (LWS) receptors, allowing for trichromatic colour vision. In some nymphalid butterfly species additional types of ommatidia contain red-sensitive receptors, which provide additional inhibitory input to green-sensitive receptors, potentially allowing for tetrachromatic colour vision in these species [43]. These recent findings are even more exciting as very similar inhibitory mechanisms on the receptor level have also been found in vertebrates (for comparisons, see [42,71]) pointing towards a common neural solution for colour coding.

How colour coding works in the retinae of stomatopod crustaceans with up to 12 different spectral sensitivities in photoreceptors in the midband rows of their eyes, remains an open question. The similarities in the structure of the neural substrate [20,73,74] and the presence of a high number of spectrally diverse photoreceptors in some butterfly species [44,48] indicate that colour coding in the arthropod subphylum of pancrustaceans may share the same basic mechanisms of neural colour coding.

For chelicerates, the situation still needs to be resolved. In jumping spiders, probably the best-studied colour-seeing clade in chelicerates, receptors from the four layers of photoreceptors, which have different spectral sensitivities, project to four different regions of their first optic ganglion, with no sign of direct inhibition, making it likely that colour processing happens at higher—hitherto unknown—brain regions [75].

In insects, a sub-group of photoreceptors in each ommatidium project to the first optic neuropil, the lamina (as short visual fibres, svf), while the remaining receptors project directly to the second optical neuropil, the medulla (as long visual fibres, lvf). Spectral opponency has been recorded in lamina neurons (e.g. [57,69,76]) even though not all spectral receptor types have terminations in this neuropile. However, the medulla has been assumed to take a more important role in colour coding, as this is where signals from both svf receptors (via lamina interneurons) and lvf receptors (directly) feed into colour-opponent neurons (e.g. [77–79]). Colour-opponent neurons have been also described in the third optic neuropil, the lobula complex (e.g. [80]). From here information reaches higher visual centres including mushroom bodies, the anterior optic tubercle and central complex (e.g. [78,81–84]), but the circuits and projections are not yet fully understood (for reviews, see [14,42,52,66]). Given recent advances in the functional understanding of brain structures with genetic tools and physiological recordings, in combination with behavioural assays, the processing of chromatic information in the optic lobes is presently best described in *Drosophila* (reviewed by Schnaitmann *et al*. [85]).

Even though colour information is most importantly used for phototaxis (e.g. [12]) and for object detection (see, for instance, [86]), it can also serve other tasks (e.g. dung beetle navigation: [87,88]; butterfly motion vision: [89]). Accordingly, colour-sensitive neurons and pathways have been found in multiple central brain regions including the mushroom bodies (e.g. [82,90]), the anterior optic tubercle (e.g. [91,92]) and the central complex (e.g. [66] and references therein), and most recently, the motion pathway of butterflies [93]. Conserved, not only among arthropods, but probably in a vast majority of colour vision systems, are also the mechanisms on the receptor and neuronal level that allow for colour constancy, the ability to recognize object colours under spectrally different illuminations [94].

## 6. Diversity of colour-guided behaviour and colour ecology

The ecological diversity of arthropods is reflected by their behavioural use of colour information and their colour ecology, as has been studied in different groups (e.g. [14]). Arthropods occur in a wide range of aquatic and terrestrial habitats and thus, in very different colour worlds. Generally, as water absorbs and scatters light of different wavelengths to different degrees [7], the demands on colour vision are high, and animals such as some stomatopod crustaceans can change the tuning of the photoreceptors depending on light habitat [20]. Nocturnal or deep-sea species are often colour-blind but there are exceptions. For instance, large flower-visiting insects can discern colour even in starlight, as summarized by Warrant & Somanathan [95]. Specifically for nocturnal species, the increasing degree of artificial light pollution at night may pose challenges for their colour vision which may impair pollination (e.g. [96,97]).

Similar to other animals, arthropods use colour information for two major purposes: the choice of a suitable light habitat by phototaxis and reliable detection, discrimination and recognition of relevant objects, where the latter includes innate colour preference as well as learned colour choice [86,98,99].

Phototaxis does not require high resolution and is therefore found in many more species which may have little other use of colour. Water fleas of the genus *Daphnia* were the first arthropods, for which habitat choice was proved to depend on the chromatic aspect of light, as they prefer light habitats with a high intensity of long-wavelength light to habitats with overall high intensity [12]. This is in contrast to colour-blind phototaxis as found, for instance, in the sister phylum, Onychophora [24,100]. Colour-guided habitat choices are common among caterpillars before pupation (e.g. [101]), and preference for light of long wavelengths is found in many herbivorous pest insects, which either feed or oviposit on green leaves [102,103]. López Reyes *et al.* [62] review what is presently known for thrips (the insect order Thysanoptera), and Döring & Kirchner [104] provide a model explaining the choice of aphids (Hemiptera) for substrates appearing green or yellow to the human eye.

Studies of colour-guided object detection and discrimination by arthropods have long focused on flowers and pollinators (e.g. [9]), which is still an important field (e.g. [105–107]). In addition, many other object classes are now known to be detected and recognized by arthropods using colour. These include conspecifics, specifically mates (e.g. in fiddler crabs: [108]; and probably in stomatopods: [109]; jumping spiders: [110]; and butterflies: [111]; fireflies: [112,113]), as well as food unrelated to flowers (e.g. in stomatopods: [114]), leaves as oviposition substrates (e.g. [103,115]) and landmarks (e.g. [116,117]; for reviews see [86,118]).

Many objects and substrates are not uniformly coloured. The pigmentation of flower petals, for instance, often varies within and between individual flowers, changing chromatic contrast (e.g. [10]) and influencing detectability (e.g. [119]). Patterns of multiple colours are found in flowers with nectar guides [99] and the bodies and appendages of animals such as mantis shrimps [20], fiddler crabs [108], spiders [120] or butterflies (e.g. [111]). Probably, such colour patterns have evolved to facilitate learning, recognition and communication. Analysing the behaviour of animals towards colour patterns may help to better understand the processing of context and natural scenes, in which objects are viewed [94], or more broadly reveal, which aspects of coloration are important given the low resolution of arthropod eyes [99].

It is currently unknown whether and how arthropods distinguish between dimensions in perceptual colour space that would be equivalent to human colour dimensions of hue, brightness and saturation (for discussions, see [52,86,121]). These questions can only be studied in animals such as honeybees or *Papilio* butterflies, for which sufficient data on photoreceptors are available (e.g. [93,94,99]) and established models, such as the Receptor Noise Limited Model [122,123] appropriately describe psychophysical performances. How colour-coded information is integrated in the brain with other visual functions is another fascinating and little explored topic.

Only quite recently, and taking many researchers by surprise, it has become obvious that arthropods may rely on colour signals when performing tasks which are classically considered colour-blind, such as motion vision [89,93,124] or skylight navigation [87,88].

## 7. Outlook: diverse colour vision requires more and diverse studies

This brief overview highlights how much more research is needed to fill in the many missing pieces of this large puzzle. Diverse colour vision requires a diversity of study tools including genetics, anatomy, physiology and importantly, behavioural tests. In this special issue Lienard *et al*. [31] review bioinformatic approaches specifically well suited to gain a better understanding of opsin diversity in arthropods. Ultimately, the hallmark is the behavioural evidence for colour perception—to demonstrate how colour information is used to guide behaviour. It continues to be challenging and laborious to conduct appropriate tests for demonstrating colour discrimination and colour constancy, and even more for showing that an animal can ignore achromatic cues. If the photoreceptor sensitivities are known, behavioural methods, such as described by Cheney *et al.* [125], Christenson *et al*. [126], Hempel de Ibarra *et al*. [99] and Werner [94], allow experimenters to design visual stimuli that are well suited to map the colour space of a species and allow for a better understanding of their perceptual colour worlds.

## Data Availability

This article has no additional data.
